# Supplementary figures and images for: An automated high-content screening and assay platform for the analysis of spheroids at subcellular resolution
Source: PLoS One. 2024 Nov 12;19(11):e0311963. doi: 10.1371/journal.pone.0311963 (PMC11556727; doi:10.1371/journal.pone.0311963)

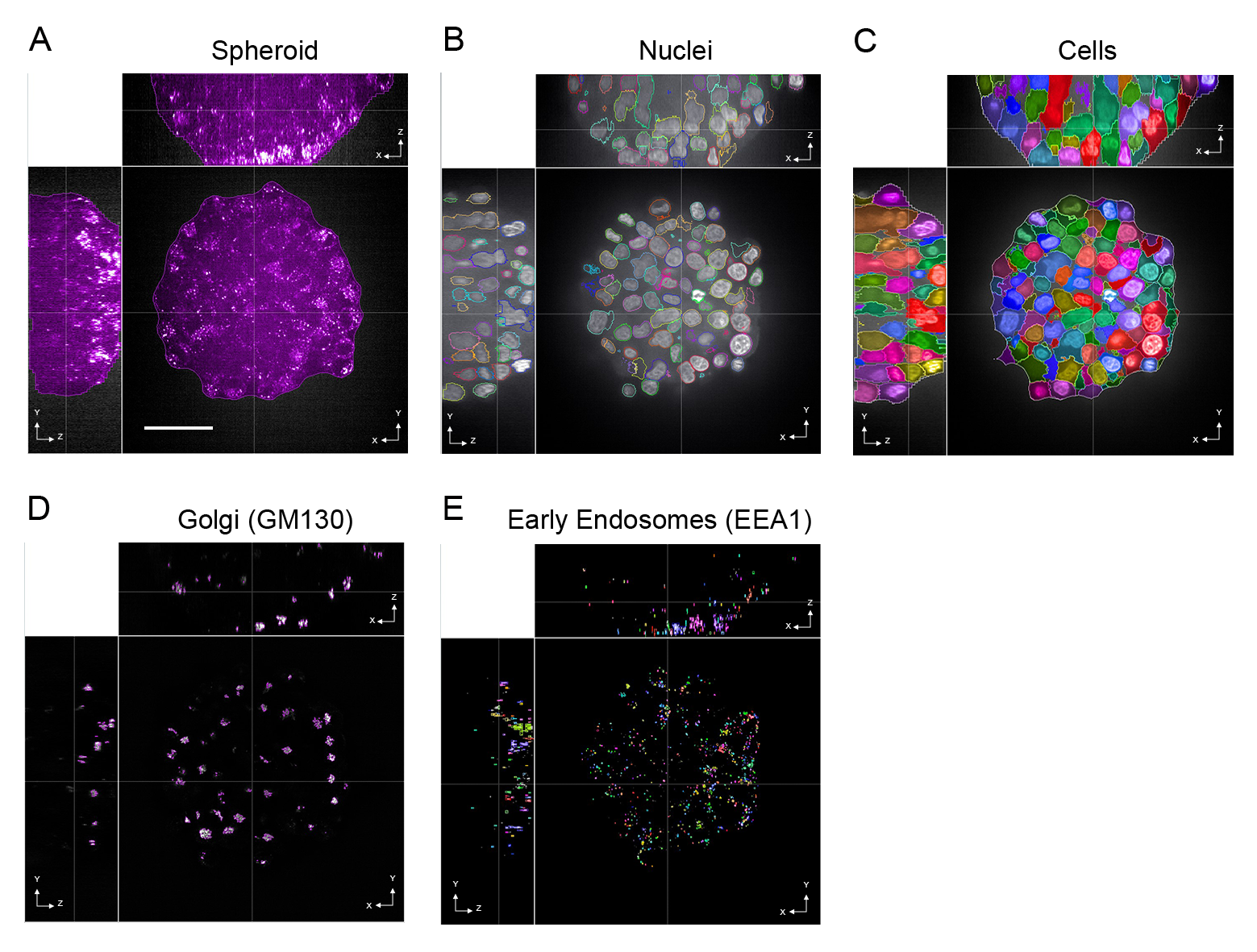

Supplement: S1 Fig — Example showing how spheroids were volumetrically analysed using Harmony software. Example segmentations of A) whole spheroid, B) individual nuclei, C) cytoplasm, D) Golgi (GM130) and E) early endosomes (EEA1). Pseudocolouring is used to highlight individual objects. Scale bar: 50 μm. (TIF) [file pone.0311963.s001.tif]

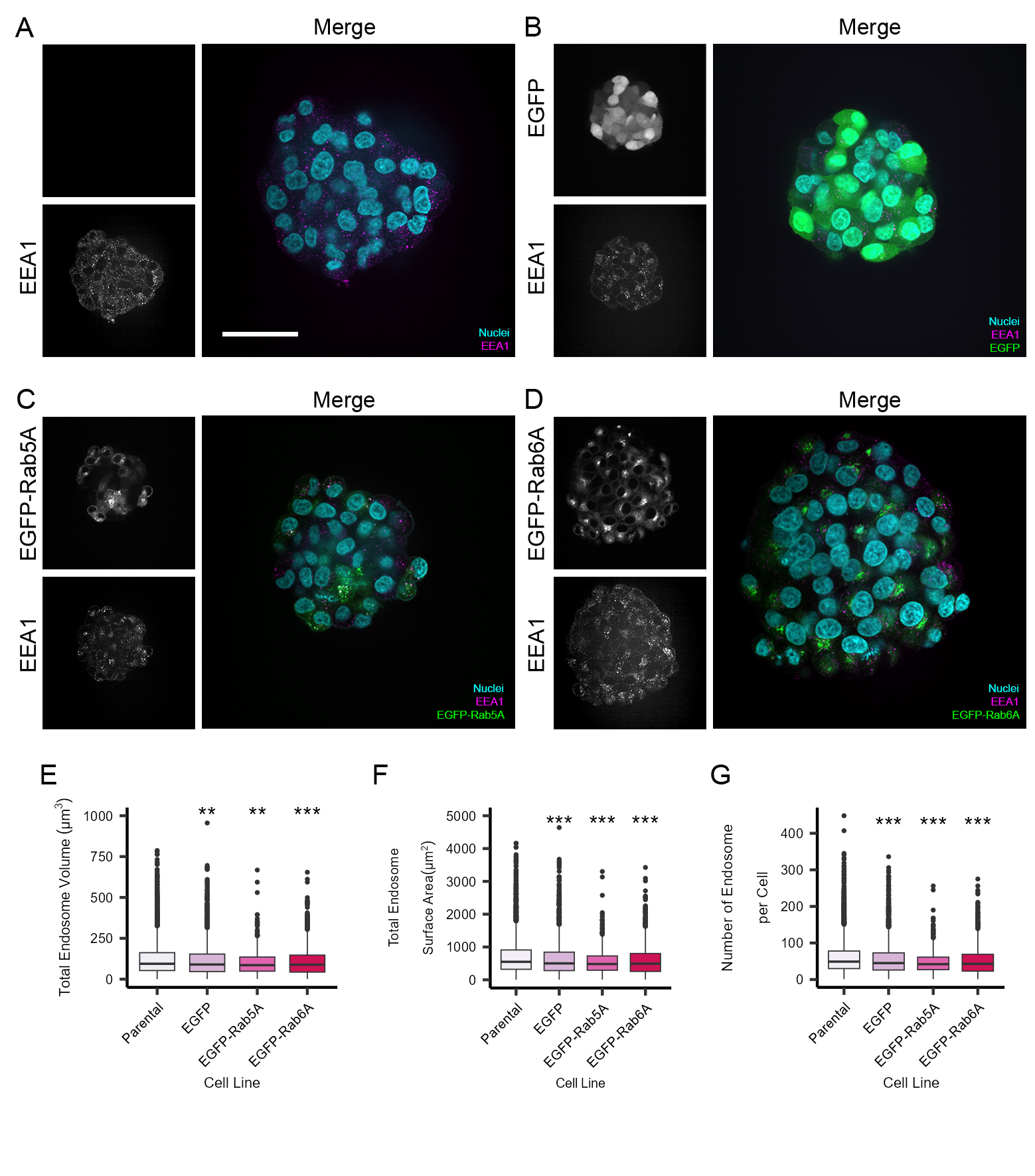

Supplement: S2 Fig — Cells were grown on a CYTOO DC45-P300-FN plate for three days to generate spheroids. The spheroids were fixed and stained with Hoechst 33342 (nuclei), and anti-EEA1 antibodies were used to immunostain the early endosomes. Images were acquired with an Opera Phenix High-Content Screening microscope using a 63x/1.15 NA water immersion objective. A-D) Representative images of the spheroids generated from the various cell lines. Scale bar: 50 μm. E) Mean total endosome volume per cell. F) Mean total endosome surface area per cell. G) Mean number of endosomes per cell. The box plots show per cell measurements indicating the median values and 50 percentiles of the data. Number of cells analysed were 3707 (parental), 2437 (EGFP), 532 (EGFP-Rab5A), and 1304 (EGFP-Rab6A) from a minimum of 12 spheroids per cell line. Data are from three technical replicate wells for each cell line. An unpaired Wilcoxon test was performed. ** indicates p<0.01 and *** indicates p<0.001. (TIF) [file pone.0311963.s002.tif]

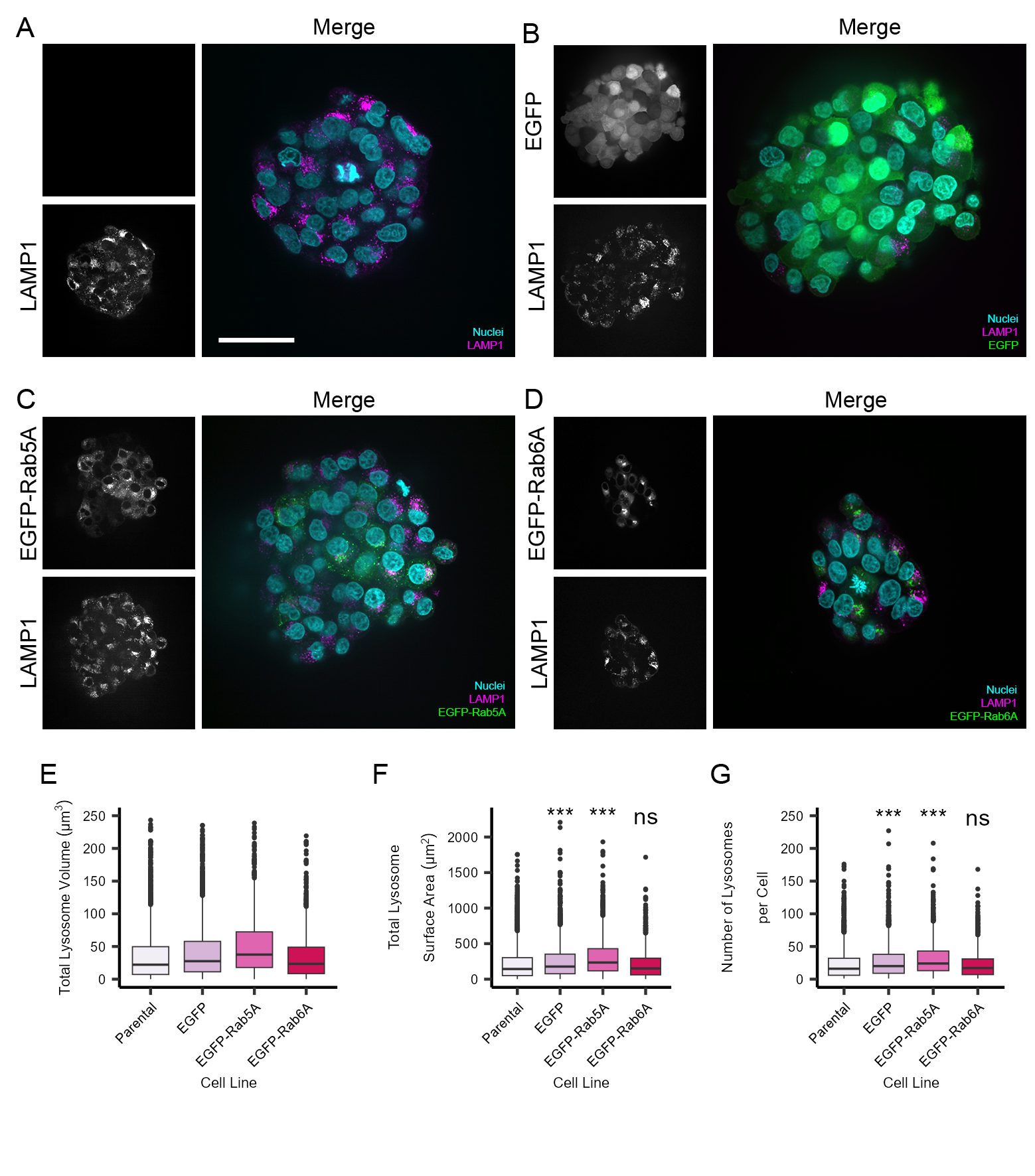

Supplement: S3 Fig — Cells were grown on a CYTOO DC45-P300-FN plate for three days to generate spheroids. The spheroids were fixed and stained with Hoechst 33342 (nuclei), and anti-LAMP1 antibodies were used to immunostain the lysosomes. Images were acquired with an Opera Phenix High-Content Screening microscope using a 63x/1.15 NA water immersion objective. A-D) Representative images of the spheroids generated from the various cell lines. Scale bar: 50 μm. E) Mean total lysosome volume per cell. F) Mean total lysosome surface area per cell. G) Mean number of lysosomes per cell. The box plots show per cell measurements indicating the median values and 50 percentiles of the data. Number of cells analysed were 3346 (parental), 2502 (EGFP), 1536 (EGFP-Rab5A), and 1143 (EGFP-Rab6A) from a minimum of 20 spheroids per cell line. Data are from three technical replicate wells for each cell line. An unpaired Wilcoxon test was performed. ns indicates not significant and *** indicates p<0.001. (TIF) [file pone.0311963.s003.tif]

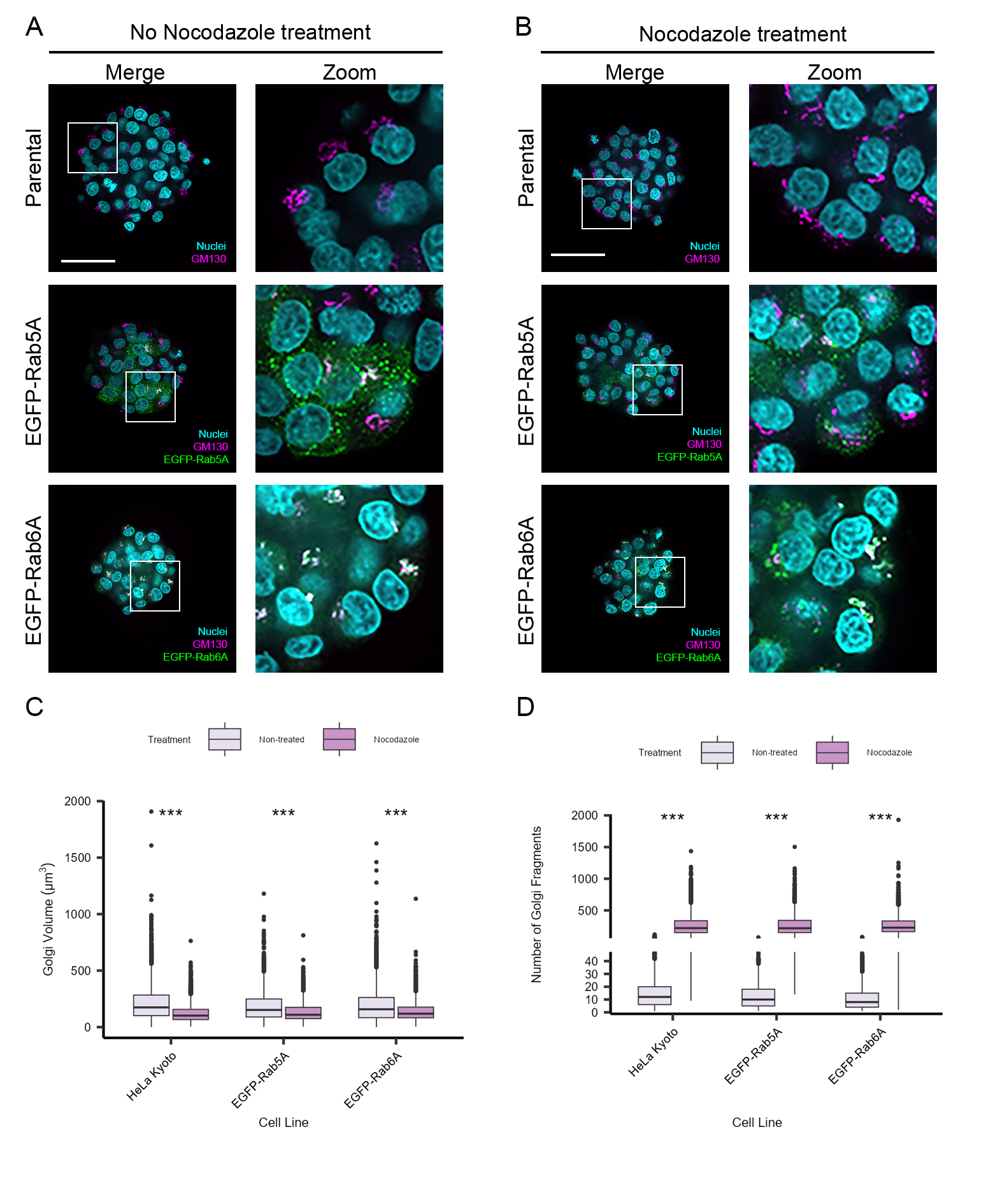

Supplement: S4 Fig — After three days of growth in a CYTOO DC45-P300-FN plate, spheroids were treated with 7.5 μM nocodazole for 30 min at 37°C. Spheroids were fixed and stained with Hoechst 33342 (nuclei), and antibodies were used to immunostain GM130 (Golgi). Images were acquired with an Opera Phenix High-Content Screening microscope with the 63x/1.15 NA water immersion objective. A) Representative images of untreated treated spheroids. Scale bar: 50 μm. B) Representative images of nocodazole treated spheroids. Scale bar: 50 μm. C) Mean Golgi volume for untreated and treated cells grown as spheroids for the various cell lines. D) Number of Golgi fragments for untreated and treated cells grown as spheroids for the various cell lines. The box plots represent per cell measurements. Number of cells analysed were 2923 (parental, non-treated), 2684 (parental, nocodazole-treated), 1994 (EGFP-Rab5A, non-treated), 1951 (EGFP-Rab5A, nocodazole-treated), 2470 (EGFP-Rab6A, non-treated) and 1897 (EGFP-Rab6A, nocodazole-treated) from a minimum 37 spheroids per cell line. Data are from two biological replicates. An unpaired Wilcoxon test was performed. *** indicates p<0.001. (TIF) [file pone.0311963.s004.tif]

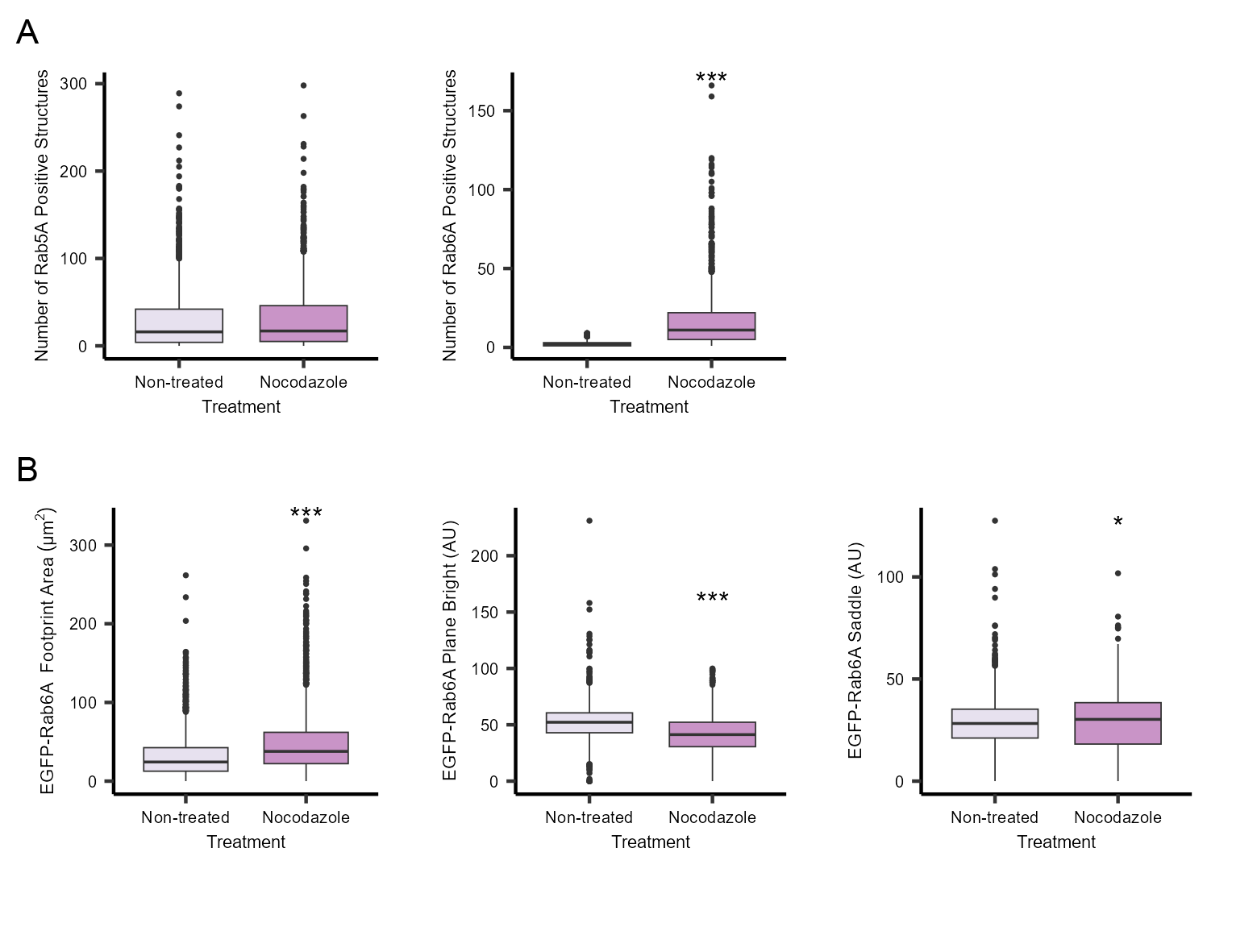

Supplement: S5 Fig — Spheroids were grown for three days in a CYTOO DC45-P300-FN plate. Spheroids were treated with 7.5 μM nocodazole for 30 min at 37°C. Then spheroids were fixed and stained with Hoechst 33342 (nuclei), and antibodies were used to immunostain GM130 (Golgi). After image acquisition, a volumetric analysis was performed. A) Quantification of the EGFP-Rab5A and EGFP-Rab6A positive structures. B) EGFP-Rab6A footprint area and texture (plane bright and saddle) measurements. The box plots represent per cell measurements. The number of cells analysed were 2016 (EGFP-Rab5A, non-treated), 1957 (EGFP-Rab5A, nocodazole-treated), 2066 (EGFP-Rab6A, non-treated) and 1799 (EGFP-Rab6A, nocodazole-treated) from a minimum of 41 spheroids per cell line. Data are from two biological replicates. An unpaired Wilcoxon test was performed. * indicates p<0.05 and *** indicates p<0.001. (TIF) [file pone.0311963.s005.tif]
