# Supplementary material for: An automated high-content screening and assay platform for the analysis of spheroids at subcellular resolution
Source: PLoS One. 2024 Nov 12;19(11):e0311963. doi: 10.1371/journal.pone.0311963 (PMC11556727; doi:10.1371/journal.pone.0311963)
Supplement: S1 Table — Analysis pipeline describes the building blocks and the thresholds used to segment spheroids, cells, Golgi, endosomes and lysosomes. The building block in orange ‘Select Population’ is used only in parental cells to give parental and GFP cells a consistent population name. The building blocks in green were used to select EGFP-expressing cells. The ‘Filter Image (2)’ and ‘Find Image Region (3)’ building blocks provide endosome (light blue) and lysosome (purple) specific thresholds. (PDF) [file pone.0311963.s006.pdf]

**Input Image**	
**Input**	Flatfield Correction: None<br>Stack Processing: 3D Analysis<br>Min. Global Binning: Dynamic
**Find Image Region**	
**Input**	Channel: Alex 647<br>ROI: None
**Method**	Method: Local Threshold<br>Threshold: 0.15<br>Region Scale: 50 µm<br>Closing: 12 µm<br>Filling: Fill Plane-Wise<br>Volume: > 3500 µm^3
**Output**	Output Population: Spheroid<br>Output Region: Spheroid
**Find Nuclei**	
**Input**	Channel: Hoechst 33342 – extended<br>ROI: Spheroid<br>ROI Region: Spheroid
**Method**	Method: C<br>Common Threshold: 0.25<br>Volume: >120 µm^3<br>Splitting Coefficient: 5<br>Individual Threshold: 0.3<br>Contrast: > -0.1<br>Accuracy/Speed: Standard/Standard
**Output**	Output Population: Nuclei
**Calculate Morphology Properties**	
**Input**	Population: Spheroid<br>Region: Spheroid
**Method**	Method: Standard<br>Volume<br>Sphericity
**Output**	Property Prefix: Spheroid
**Calculate Properties**	
**Input**	Population: Spheroid
**Method**	Method: By Related Population<br>Related Population: Nuclei<br>Number of Nuclei
**Output**	Property Suffix: per Spheroid

**Select Region**	
**Input**	Population: Nuclei<br>Region: Nucleus
**Method**	Method: Standard<br>Inner Center
**Output**	Region Prefix: Nucleus
**Calculate Position Properties**	
**Input**	Population: Nuclei<br>Region: Nucleus Inner Center
**Method**	Method: Standard<br>Nearest Neighbour Distance
**Output**	Property Prefix: Nucleus Inner Center
**Find Cytoplasm**	
**Input**	Channel: Hoechst 33342 – extended<br>Nuclei: Nuclei
**Method**	Method: A<br>Individual Threshold: 0.01<br>Restrictive Region: Spheroid<br>Accuracy/Speed: Standard/Standard
**Select Population**	
**Input**	Population: Nuclei
**Method**	Method: Common Filters<br>Remove Objects: None<br>Region: Cell
**Output**	Output Population: Cells
**Calculate Intensity Properties**	
**Input**	Channel: Alexa 488<br>Population: Nuclei<br>Region: Cell
**Method**	Method: Standard<br>Mean
**Output**	Property Prefix: Intensity Cell Alexa 488
**Select Population**	
**Input**	Population: Nuclei
**Method**	Method: Filter by Property<br>Intensity Cell Alexa 488 Mean: > 130 or 150
**Output**	Output Population: Cells
**Filter Image**	
**Input**	Channel: Alexa 568
**Method**	Method: Smoothing

	Filter: Gaussian<br>Width: 40 px
**Output**	Output Image: Gaussian Smoothed
**<u>Calculate Image</u>**	
**Method**	Method: By Formula<br>Formula: A/B<br>Channel A: Alexa 568<br>Channel B: Gaussian Smoothed<br>Negative Values: Set to Zero<br>Undefined Values: Set to Zero
**Output**	Output Image: Calculated Image
**<u>Find Image Region (2)</u>**	
**Input**	Channel: Calculated Image<br>ROI: Cells<br>ROI Region: Cell
**Method**	Method: Absolute Threshold<br>Lowest Intensity: ≥ 2<br>Highest Intensity: ≤ INF<br>Volume: > 2 µm^3
**Output Population**	Output Population: Golgi<br>Output Region: Golgi
**<u>Filter Image (2)</u>**	
**Input**	Channel: Alexa 647
**Method**	Method: Texture PLS<br>ROI: Cells<br>ROI Region: Cell<br>Filter: Sport Bright<br>Scale XY: 0.65/ 0.48 µm<br>Scale Z: 0.1 µm<br>PSF Aspect Ratio: 2
**Output**	Output Image: Spot Bright
**<u>Find Image Region (3)</u>**	
**Input**	Channel: Sport Bright<br>ROI: Cells<br>ROI Region: Cell
**Method**	Method: Local Threshold<br>Threshold: 0.1/0.2<br>Region Scale: 1 µm<br>Smoothing: 0.2 µm<br>Volume: > 1 µm^3

**Output**	Output Population: Endosomes/Lysosomes<br>Output Region: Endosomes/Lysosomes
**Calculate Morphology Properties (2)**	
**Input**	Population: Cell<br>Region: Endosomes/Lysosomes
**Method**	Method: Standard<br>Volume<br>Surface Area<br>Sphericity
**Output**	Property Prefix: Endosomes/Lysosomes
**Calculate Morphology Properties (3)**	
**Input**	Population: Cells<br>Region: Golgi
**Method**	Method: Standard<br>Volume<br>Surface Area<br>Number of Fragments<br>Sphericity
**Output**	Property Prefix: Golgi
**Calculate Properties (2)**	
**Input**	Population: Cells
**Method**	Method: By Related Population<br>Related Population: Endosomes/Lysosomes<br>Number of Endosomes/Lysosomes
**Output**	Property Suffix: per Cell
**Define Results**	
**Results**	Method: List of Output<br>Population: Nuclei<br>Nucleus Inner Center Nearest Neighbour Distance [µm]: Mean<br><br>Population: Golgi<br>Number of Objects<br><br>Population: Endosomes/Lysosomes<br>Number of Objects<br><br>Population: Cells<br>Number of Objects<br>Apply to All: Mean<br>Golgi Volume [μm^3]: Mean

	Golgi Surface Area [µm^2]: Mean<br>Golgi Sphericity: Mean<br>Golgi Number of Fragments: Mean<br>Endosomes/Lysosomes Volume [µm^3]: Mean<br>Endosomes/Lysosomes Surface Area [µm^2]: Mean<br>Endosomes/Lysosomes Sphericity: Mean<br>Number of Endosomes/Lysosomes – per Cell: Mean<br><br>Population: Spheroid<br>Number of Objects<br>Apply to All: Mean<br>Spheroid Volume [µm^3]: Mean<br>Spheroid Surface Area [µm^2]: Mean<br>Spheroid Sphericity: Mean<br>Number of Nuclei – per Spheroid: Mean<br><br>Object Results:<br>Population: Nuclei: Use Selected Well Results<br>Population: Golgi: Use Selected Well Results<br>Population: Cells: Use Selected Well Results<br>Population: Endosomes/Lysosomes: Use Selected Well Results<br>Population: Spheroid: Use Selected Well Results
