# Supplementary material for: An automated high-content screening and assay platform for the analysis of spheroids at subcellular resolution
Source: PLoS One. 2024 Nov 12;19(11):e0311963. doi: 10.1371/journal.pone.0311963 (PMC11556727; doi:10.1371/journal.pone.0311963)
Supplement: S3 Table — Analysis pipeline describes the building blocks and the thresholds used to segment spheroids, cells and Golgi. The building block in orange ‘Select Population’ is used only in parental cells to give parental and GFP cells a consistent population name. The building blocks in green were used to select EGFP-expressing cells. (PDF) [file pone.0311963.s008.pdf]

**Input Image**	
**Input**	Flatfield Correction: None<br>Stack Processing: 3D Analysis<br>Min. Global Binning: Dynamic
**Find Image Region**	
**Input**	Channel: Alex 647<br>ROI: None
**Method**	Method: Local Threshold<br>Threshold: 0.15<br>Region Scale: 10 µm<br>Closing: 12 µm<br>Filling: Fill Plane-Wise<br>Volume: > 3500 μm^3
**Output**	Output Population: Spheroid<br>Output Region: Spheroid
**Find Nuclei**	
**Input**	Channel: Hoechst 33342 – extended<br>ROI: Spheroid<br>ROI Region: Spheroid
**Method**	Method: C<br>Common Threshold: 0.25<br>Volume: >120 μm^3<br>Splitting Coefficient: 5<br>Individual Threshold: 0.3<br>Contrast: > -0.1<br>Accuracy/Speed: Standard/Standard
**Output**	Output Population: Nuclei
**Find Cytoplasm**	
**Input**	Channel: Hoechst 33342 – extended<br>Nuclei: Nuclei
**Method**	Method: A<br>Individual Threshold: 0.01<br>Restrictive Region: Spheroid<br>Accuracy/Speed: Standard/Standard
**Select Population**	
**Input**	Population: Nuclei
**Method**	Method: Common Filters<br>Remove Objects: None<br>Region: Cell
**Output**	Output Population: Cells

<table>
<tr><td colspan="2">Calculate Intensity Properties</td></tr>
<tr><td>Input</td><td>Channel: Alexa 488
Population: Nuclei
Region: Cell</td></tr>
<tr><td>Method</td><td>Method: Standard
Mean</td></tr>
<tr><td>Output</td><td>Property Prefix: Intensity Cell Alexa 488</td></tr>
<tr><td colspan="2">Select Population</td></tr>
<tr><td>Input</td><td>Population: Nuclei</td></tr>
<tr><td>Method</td><td>Method: Filter by Property
Intensity Cell Alexa 488 Mean: > 150</td></tr>
<tr><td>Output</td><td>Output Population: Cells</td></tr>
<tr><td colspan="2">Filter Image</td></tr>
<tr><td>Input</td><td>Channel: Alexa 568</td></tr>
<tr><td>Method</td><td>Method: Texture PLS
ROI: Cells
ROI Region: Cell
Filter: Sport Bright
Scale XY: 0.42
Scale Z: 0.2
PSF Aspect Ratio: 2</td></tr>
<tr><td>Output</td><td>Output Image: Sport Bright</td></tr>
<tr><td>Calculate Image</td><td></td></tr>
<tr><td>Method</td><td>Method: By Formula
Formula: IIF(A> 900, A, 0)
Channel A: Alexa 568
Negative Values: Set to Zero
Undefined Values: Set to Zero</td></tr>
<tr><td>Output</td><td>Output Image: Calculated Image</td></tr>
<tr><td colspan="2">Calculate Image (2)</td></tr>
<tr><td>Method</td><td>Method: By Formula
Formula: A||B
Channel A: Spot Bright
Channel B: Calculated Image
Negative Values: Set to Zero
Undefined Values: Set to Zero</td></tr>
<tr><td>Output</td><td>Output Image: Golgi</td></tr>
<tr><td colspan="2">Find Image Region (2)</td></tr>
<tr><td>Input</td><td>Channel: Golgi
ROI: Cells</td></tr>
</table>

	ROI Region: Cell
**Method**	Method: Absolute Threshold<br>Lowest Intensity: ≥ 1<br>Highest Intensity: ≤ INF<br>Volume: > 0.1 µm^3
**Output Population**	Output Population: Golgi<br>Output Region: Golgi
**<u>Calculate Morphology Properties</u>**	
**Input**	Population: Cells<br>Region: Golgi
**Method**	Method: Standard<br>Volume<br>Surface Area<br>Number of Fragments<br>Sphericity<br>Footprint Area
**Output**	Property Prefix: Golgi
**<u>Define Results</u>**	
**Results**	Method: List of Output<br>Population: Nuclei<br><br>Population: Golgi<br>Number of Objects<br><br>Population: Cells<br>Number of Objects<br>Apply to All: Mean<br>Golgi Volume [µm^3]: Mean<br>Golgi Surface Area [µm^2]: Mean<br>Golgi Sphericity: Mean<br>Golgi Number of Fragments: Mean<br>Golgi Footprint Area [µm]: Mean<br><br>Population: Spheroid<br>Number of Objects<br><br>Object Results:<br>Population: Nuclei: None<br>Population: Golgi: Use Selected Well Results<br>Population: Cells: Use Selected Well Results<br>Population: Spheroid: Use Selected Well Results
