# Supplementary material for: An automated high-content screening and assay platform for the analysis of spheroids at subcellular resolution
Source: PLoS One. 2024 Nov 12;19(11):e0311963. doi: 10.1371/journal.pone.0311963 (PMC11556727; doi:10.1371/journal.pone.0311963)
Supplement: S4 Table — Analysis pipeline describes the building blocks and the thresholds used to segment spheroids, cells and EGFP-Rab5A-positive structures. (PDF) [file pone.0311963.s009.pdf]

**Input Image**	
**Input**	Flatfield Correction: None<br>Stack Processing: 3D Analysis<br>Min. Global Binning: Dynamic
**Find Image Region**	
**Input**	Channel: Alex 647<br>ROI: None
**Method**	Method: Local Threshold<br>Threshold: 0.15<br>Region Scale: 10 µm<br>Closing: 12 µm<br>Filling: Fill Plane-Wise<br>Volume: > 3500 µm^3
**Output**	Output Population: Spheroid<br>Output Region: Spheroid
**Find Nuclei**	
**Input**	Channel: Hoechst 33342 – extended<br>ROI: Spheroid<br>ROI Region: Spheroid
**Method**	Method: C<br>Common Threshold: 0.25<br>Volume: >120 µm^3<br>Splitting Coefficient: 5<br>Individual Threshold: 0.3<br>Contrast: > -0.1<br>Accuracy/Speed: Standard/Standard
**Output**	Output Population: Nuclei
**Find Cytoplasm**	
**Input**	Channel: Hoechst 33342 – extended<br>Nuclei: Nuclei
**Method**	Method: A<br>Individual Threshold: 0.01<br>Restrictive Region: Spheroid<br>Accuracy/Speed: Standard/Standard
**Calculate Intensity Properties**	
**Input**	Channel: Alexa 488<br>Population: Nuclei<br>Region: Cell
**Method**	Method: Standard<br>Mean

**Output**	Property Prefix: Intensity Cell Alexa 488
**<u>Select Population</u>**	
**Input**	Population: Nuclei
**Method**	Method: Filter by Property<br>Intensity Cell Alexa 488 Mean: > 150
**Output**	Output Population: Cells
**<u>Calculate Image</u>**	
**Method**	Method: By Formula<br>Formula: IIF(A> 750, A, 0)<br>Channel A: Alexa 488<br>Negative Values: Set to Zero<br>Undefined Values: Set to Zero
**Output**	Output Image: Calculated Image_GFP
**<u>Find Image Region (2)</u>**	
**Input**	Channel: Calculated Image_GFP<br>ROI: Cells<br>ROI Region: Cell
**Method**	Method: Absolute Threshold<br>Lowest Intensity: ≥ 750<br>Highest Intensity: ≤ INF<br>Volume: > 0.2 μm^3
**Output Population**	Output Population: EGFP-Rab5a<br>Output Region: EGFP-Rab5a
**<u>Calculate Properties</u>**	
**Input**	Population: Cells
**Method**	Method: By Related Population<br>Population B: EGFP-Rab5a<br>Number of EGFP-Rab5a
**Output**	Property Prefix: per Cell
**<u>Define Results</u>**	
**Results**	Method: List of Output<br>Population: Nuclei<br><br>Population: Cells<br>Number of Objects<br>Number of EGFP-Rab5a – per Cell: Mean<br><br>Population: Spheroid<br>Number of Objects

	Population: EGFP-Rab5a<br>Number of Objects<br><br>Object Results:<br>Population: Nuclei: None<br>Population: EGFP-Rab5a: Use Selected Well Results<br>Population: Spheroid: Use Selected Well Results<br>Population: Cells: Use Selected Well Results
