# Supplementary material for: An automated high-content screening and assay platform for the analysis of spheroids at subcellular resolution
Source: PLoS One. 2024 Nov 12;19(11):e0311963. doi: 10.1371/journal.pone.0311963 (PMC11556727; doi:10.1371/journal.pone.0311963)
Supplement: S5 Table — Analysis pipeline describes the building blocks and the thresholds used to segment spheroids, cells and EGFP-Rab6A-positive structures. Building blocks ‘Calculate Image’ and ‘Find Image region (2)’ contain specific values for non-treated, BFA (purple)- and nocodazole-treated (light blue) EGFP-Rab6A cells. (PDF) [file pone.0311963.s010.pdf]

**Input Image**	
**Input**	Flatfield Correction: None<br>Stack Processing: 3D Analysis<br>Min. Global Binning: Dynamic
**Find Image Region**	
**Input**	Channel: Alex 647<br>ROI: None
**Method**	Method: Local Threshold<br>Threshold: 0.15<br>Region Scale: 10 µm<br>Closing: 12 µm<br>Filling: Fill Plane-Wise<br>Volume: > 3500 $µm^3$
**Output**	Output Population: Spheroid<br>Output Region: Spheroid
**Find Nuclei**	
**Input**	Channel: Hoechst 33342 – extended<br>ROI: Spheroid<br>ROI Region: Spheroid
**Method**	Method: C<br>Common Threshold: 0.25<br>Volume: >120 $µm^3$<br>Splitting Coefficient: 5<br>Individual Threshold: 0.3<br>Contrast: > -0.1<br>Accuracy/Speed: Standard/Standard
**Output**	Output Population: Nuclei
**Find Cytoplasm**	
**Input**	Channel: Hoechst 33342 – extended<br>Nuclei: Nuclei
**Method**	Method: A<br>Individual Threshold: 0.01<br>Restrictive Region: Spheroid<br>Accuracy/Speed: Standard/Standard
**Calculate Intensity Properties**	
**Input**	Channel: Alexa 488<br>Population: Nuclei<br>Region: Cell
**Method**	Method: Standard<br>Mean

**Output**	Property Prefix: Intensity Cell Alexa 488
**Select Population**	
**Input**	Population: Nuclei
**Method**	Method: Filter by Property<br>Intensity Cell Alexa 488 Mean: > 150
**Output**	Output Population: Cells
**Calculate Image**	
**Method**	Method: By Formula<br>Formula: IIF(A> 4000/2500/quantile(A, 0.8).quantile, A, 0)<br>Channel A: Alexa 488<br>Negative Values: Set to Zero<br>Undefined Values: Set to Zero
**Output**	Output Image: Calculated Image_GFP
**Find Image Region (2)**	
**Input**	Channel: Calculated Image_GFP<br>ROI: Cells<br>ROI Region: Cell
**Method**	Method: Absolute Threshold<br>Lowest Intensity: ≥ 750<br>Highest Intensity: ≤ INF<br>Smoothing: 0.2 µm<br>Volume: > 10/0.2/0.2 μm^3
**Output Population**	Output Population: EGFP-Rab6a<br>Output Region: EGFP-Rab6a
**Calculate Texture Properties**	
**Input**	Channel: Alexa 488<br>Population: Cells<br>Region: EGFP-Rab6a
**Method**	Method: PLS Feature<br>Filter: Plane Bright<br>Scale XY: 0.01 µm<br>Scale Z: 1 µm<br>PSF Aspect Ratio: 2
**Output**	Property Prefix: EGFP-Rab6a
**Calculate Texture Properties (2)**	
**Input**	Channel: Alexa 488<br>Population: Cells<br>Region: EGFP-Rab6a
**Method**	Method: PLS Feature<br>Filter: Saddle

	Scale XY: 0.1 µm<br>Scale Z: 1 µm<br>PSF Aspect Ratio: 2
**Output**	Property Prefix: EGFP-Rab6a
**Calculate Morphology Properties**	
**Input**	Population: Cells<br>Region: EGFP-Rab6a
**Method**	Method: Standard<br>Volume<br>Footprint Area
**Output**	Property Prefix: EGFP-Rab6a
**Calculate Properties**	
**Input**	Population: Cells
**Method**	Method: By Related Population<br>Population B: EGFP-Rab6a<br>Number of EGFP-Rab6a
**Output**	Property Prefix: per Cell
**Define Results**	
**Results**	Method: List of Output<br>Population: Nuclei<br><br>Population: Cells<br>Number of Objects<br>EGFP-Rab6a Plane Bright 0.107991360691 µm: Mean<br>EGFP-Rab6a Saddle 1.07991360691 µm: Mean<br>EGFP-Rab6a Volume [$µm^3$]: Mean<br>EGFP-Rab6a Footprint Area [$µm^2$]: Mean<br>Number of EGFP-Rab6a – per Cell: Mean<br><br>Population: Spheroid<br>Number of Objects<br><br>Population: EGFP-Rab6a<br>Number of Objects<br><br>Object Results:<br>Population: Nuclei: None<br>Population: EGFP-Rab6a: Use Selected Well Results<br>Population: Spheroid: Use Selected Well Results<br>Population: Cells: Use Selected Well Results
