# Supplementary material for: An automated high-content screening and assay platform for the analysis of spheroids at subcellular resolution
Source: PLoS One. 2024 Nov 12;19(11):e0311963. doi: 10.1371/journal.pone.0311963 (PMC11556727; doi:10.1371/journal.pone.0311963)
Supplement: S6 Table — Analysis pipeline describes the building blocks and thresholds used to segment spheroids as well as the building block ‘Determine Well Layout’ for the rescan with the 63x water immersion objective. (PDF) [file pone.0311963.s011.pdf]

Input Image	
**Input**	Flatfield Correction: None<br>Stack Processing: Individual Planes<br>Create Global Image<br>Min. Global Binning: Dynamic
**Find Image Region**	
**Input**	Channel: Hoechst 33342-extended (global)<br>ROI: Image Area (global)<br>ROI Region: Image Area
**Method**	Method: Common Threshold<br>Threshold: 0.13<br>Split into Objects<br>Area: > 100 µm^2<br>Fill Holes
**Output**	Output Population: Spheroid<br>Output Region: Spheroid
**Calculate Morphology Properties**	
**Input**	Population: Spheroid<br>Region: Spheroid
**Method**	Method: Standard<br>Area<br>Roundness<br>Width
**Output**	Property Prefix: Spheroid
**Select Population**	
**Input**	Population: Spheroid
**Method**	Method: Filter by Property<br>Spheroid Roundess: > 0.25<br>Spheroid Area [µm^2]: > 3000<br>Spheroid Width [µm]: > 35.7<br>Spheroid Width [µm]: < 150<br>Spheroid Area [µm^2]: < 20000<br>Boolean Operations: F1 and F2 and F3 and F4 and F5
**Output**	Output Population: Spheroid 2

Determine Well Layout	
**Input**	Population: Spheroid 2<br>Region: Spheroid
**Method**	Method: XY<br>Rescan Magnification: 63x<br>Max No of Fields: 10

<table>
<tr><td></td><td>Object Margin: 10 µm
Field Overlap: 10%</td></tr>
<tr><td>Output</td><td>Output Population: Well Layout (global)</td></tr>
<tr><td colspan="2"><u>Define Results</u></td></tr>
<tr><td>Results</td><td>Method: List of Output
Population: Spheroid

Population: Spheroid 2
Number of Objects
Spheroid Area [µm^2]: Mean
Spheroid Roundness: Mean
Spheroid Width [µm]: Mean

Object Results:
Population: Spheroid: None
Population: Spheroid 2: Use Selected Well Results</td></tr>
</table>
