# Supplementary material for: An automated high-content screening and assay platform for the analysis of spheroids at subcellular resolution
Source: PLoS One. 2024 Nov 12;19(11):e0311963. doi: 10.1371/journal.pone.0311963 (PMC11556727; doi:10.1371/journal.pone.0311963)
Supplement: S7 Table — Analysis pipeline describes the building blocks and thresholds used to segment spheroids as well as what morphological features were measured. (PDF) [file pone.0311963.s012.pdf]

**Input Image**	
**Input**	Flatfield Correction: None<br>Stack Processing: Individual Planes<br>Create Global Image<br>Min. Global Binning: Dynamic
**Find Image Region**	
**Input**	Channel: Hoechst 33342-extended (global)<br>ROI: Image Area (global)<br>ROI Region: Image Area
**Method**	Method: Common Threshold<br>Threshold: 0.1<br>Split into Objects<br>Area: > 100 μm^2<br>Fill Holes
**Output**	Output Population: Spheroid<br>Output Region: Spheroid
**Calculate Morphology Properties**	
**Input**	Population: Spheroid<br>Region: Spheroid
**Method**	Method: Standard<br>Area<br>Roundness<br>Width
**Output**	Property Prefix: Spheroid
**Select Population**	
**Input**	Population: Spheroid
**Method**	Method: Filter by Property<br>Spheroid Roundess: > 0.25<br>Spheroid Area [μm^2]: > 500<br>Spheroid Width [µm]: > 70<br>Spheroid Width [µm]: < 150<br>Boolean Operations: F1 and F2 and F3 and F4
**Output**	Output Population: Spheroid 2
**Select Population 2**	
**Input**	Population: Spheroid 2
**Method**	Method: Common Filters<br>Remove Border Objects<br>Region: Spheroid
**Output**	Output Population: Spheroid 3
**Calculate Morphology Properties (2)**	

**Input**	Population: Spheroid 3<br>Region: Spheroid
**Method**	Method: Standard<br>Area<br>Roundness<br>Width
**Output**	Property Prefix: Spheroid 3
**<u>Define Results</u>**	
**Results**	Method: List of Output<br>Population: Spheroid<br>Number of Objects<br>Spheroid Area [µm^2]: Mean<br>Spheroid Roundness: Mean<br>Spheroid Width [µm]: Mean<br><br>Population: Spheroid 2<br><br>Population: Spheroid 3<br>Number of Objects<br>Spheroid Area [µm^2]: Mean<br>Spheroid Roundness: Mean<br>Spheroid Width [µm]: Mean<br><br>Object Results:<br>Population: Spheroid: Use Selected Well Results<br>Population: Spheroid 2: None<br>Population: Spheroid 3: Use Selected Well Results
