# Supplementary material for: An automated high-content screening and assay platform for the analysis of spheroids at subcellular resolution
Source: PLoS One. 2024 Nov 12;19(11):e0311963. doi: 10.1371/journal.pone.0311963 (PMC11556727; doi:10.1371/journal.pone.0311963)
Supplement: Attachment — Submitted filename: Response to Reviewers.docx [file pone.0311963.s013.docx]

**Academic Editor Comments:**

1. Please ensure that your manuscript meets PLOS ONE's style requirements, including those for file naming.

*This has been checked and our manuscript conforms to the correct style.*

2. We note that the grant information you provided in the ‘Funding Information’ and ‘Financial Disclosure’ sections do not match.

*We are not sure which elements ‘do not match’, however we believe that we have added the correct information to the relevant boxes on resubmission.*

3. Thank you for stating the following financial disclosure:
"MMM is supported by the Government of Ireland Postdoctoral Fellowship from the Irish Research Council (GOIPD/2023/1135)”. Please state what role the funders took in the study. If the funders had no role, please state: "The funders had no role in study design, data collection and analysis, decision to publish, or preparation of the manuscript."

*We have added this statement, as indeed the funders played no role in the study itself.*

4. Thank you for stating the following in the Acknowledgments Section of your manuscript:
"The authors thank all members of the lab for their input and helpful discussions. MMM is supported by the Government of Ireland Postdoctoral Fellowship from the Irish Research Council (GOIPD/2023/1135). The JCS Laboratory is supported by the UCD College of Science. " We note that you have provided funding information that is not currently declared in your Funding Statement. However, funding information should not appear in the Acknowledgments section or other areas of your manuscript.

*We have removed the funding information from the Acknowledgements as requested.*

5. In this instance it seems there may be acceptable restrictions in place that prevent the public sharing of your minimal data. However, in line with our goal of ensuring long-term data availability to all interested researchers, PLOS’ Data Policy states that authors cannot be the sole named individuals responsible for ensuring data access.

*We can provide contact information for UCD Research, the unit that oversees all research activities in the university, however in fact the PIs are a more stable form of contact than the professional staff who work in UCD Research. Also to note, UCD Research takes no responsibility for holding the research data of individual PIs. In UCD PIs are obliged to hold their own data in a responsible manner, and all PIs must complete a research integrity course that addresses this key point, and then comply with it.*

6. Please review your reference list to ensure that it is complete and correct.

*We have reviewed our reference list and believe it to be accurate. None of the cited works have been retracted.*

**Reviewer #1:**

*We thank the reviewer for their time reviewing our manuscript and we appreciate the constructive feedback. We have read the feedback carefully and have implemented their suggestions, which we believe address all the concerns raised. On the headline comment that our original manuscript lacks details that would allow others to reproduce the approach, in the revised manuscript we now provide significantly more methodological detail, and importantly we include the full image analysis pipelines used in all experiments.*

The study by Mysior and Simpson is valuable for the 3D HTM/HCA community but lacks many details that would be required to reproduce and/or scale up the approach, including the primary data. specifically, the following questions should be addressed. Plus a general answer: has the process been tested on different cell models or only in the HeLa subclone described?

*In the original optimisation experiments carried out, we worked with both HeLa Kyoto and HEK293 cells. The HeLa Kyoto cells formed spheroids in a more consistent manner than the HEK293 cells, and so these were taken forward in the study. Similar micropatterned plates have been shown (in a publication from researchers at CYTOO) to support spheroid growth based on HCT-116, T-47D, MCF-7, MDA-MB-231, A549, HT-29 and HeLa cells, although in that paper no single cell or subcellular information was acquired. These details have been added to the revised manuscript (lines 234-238).*

1. it would be useful to have more details about the plate processing - i.e., volumes, how are the plate handled (manual, robotic)

*We have now provided an expanded Materials and Methods section giving these additional details (lines 143-144, 146-147, 149-150, 153-157).*

2. were different antibody incubation times and/or triton incubation times tested?

*The antibodies used in this particular study are well known to us and required little optimisation. However, we have added a note in the revised manuscript suggesting that optimisation of other antibodies is required (lines 153-154).*

3. it is unclear if segmentation was performed on each 2D plane and then somewhat concatenated or as true 3D segmentation. why was the cytoplasm segmented off a "non specific" hoechst signal and not GFP?

*The 3D segmentation algorithms with Harmony calculate a 3D image based on the selected stack planes. The entire 3D image (image cube) is analysed instead of using single planes (or maximum projection). This information has now been added to the image analysis section of the Materials and Methods (lines 189-191). The use of residual Hoechst in the cytoplasm is a commonly used approach in image analysis to identify entire cells. It provides two key advantages in studies such as ours. Firstly, it means that only one colour channel is needed to segment individual cells and nuclei, leaving three additional channels available for more critical information (one GFP-channel and two antibody stains). Secondly, it also means that cell lines not expressing GFP-tagged proteins (for example the parental cells used in this study) can still be identified and segmented in exactly the same way as the cell lines stably expressing GFP-tagged proteins. A sentence has been added to the image analysis section acknowledging that a plasma membrane stain can also be used to segment cells and the cytoplasm (lines 189-191).*

4. more details are needed to understand why and how filters were used to segment organelles and how thresholds were chosen

*Given that the focus of this paper is more methodological, and that we intended to provide sufficient details for others to be able to replicate this approach, in the revised manuscript, we now provide the complete image analysis pipelines as tables (Tables S1 to S7) in the Appendix detailing which building blocks, filters and threshold values were used to segment spheroids, cells and organelles. Information about the analysis pipelines has been added to the Material and Methods section (lines 205-206).*

5. what is a little problematic is that somehow the authors calculate (estimate?) volumes but it is not explained how and the imaging conditions described clearly show that optical section is too big to be at Nyquist, hence the volume will not be precise. also, there is no mention of the obvious z distortion and if/how it was compensated in the calculations

*We chose this optical sectioning distance as a compromise to reduce imaging times and data volumes, given that our methodological approach is designed to be used in a screening format with potentially large numbers of plates. For the high-magnification images of spheroids a slice interval of 1 um was selected, which is indeed double the Nyquist distance (0.5 um) for this particular objective. We added a sentence in the Methods section clarifying this point (lines 177-180). However, throughout the manuscript we repeatedly acknowledge that volumetric measurements made using any form of fluorescence should be treated with caution (for example lines 463-466, 473-476, 493-495) and that rather that these data are better used in a comparative way between samples.*

6. indication of replicates (technical and biological) should be clearly spelled out in the methods section

*The specific number of replicates varies slightly between each experiment. In the original manuscript, this information was already provided in each figure legend. However, we acknowledge that this information was missing from Figure 1, and this has now been added.*

7. the statement "Single cell data could not be statistically analysed with standard statistical tests due to the high number of data values" is unacceptable as the number of single cell data values is not very high and single cell analysis is essential if the effort to segment single cells is implemented

*Statistical analysis has been performed for the data in Figures 3-5 and S2-S5. The figures have been modified to reflect this and information on how the statistical analysis was performed has been added (lines 211-214) and is in the relevant figure legends. It is important to note, however, that when comparing features for thousands of individual organelles from hundreds of cells, even small differences between values result in extremely small p-values that may be misleading. We have discussed this with an expert statistician and taken advice on how to analyse very large data sets such as this. This point is also now included in the Discussion section (lines 526-530).*

8. what is the fraction of successful spheroid formed over total seeded?

*In a typical experiment, approximately 90 % of the micropatterns contained cells 1h to 2h after cell seeding. From this, 60-80 % of the micropatterns eventually support spheroid formation and development after 3 days of growth. We added this information into the revised manuscript (lines 241-243).*

9. in Figure 2 the scale bar seems wrong, especially when compared to the other figures

*We thank the reviewer for spotting this. Indeed the scale bar was incorrectly annotated on the figure. The scale bar has been corrected, and a new Figure 2 uploaded.*

**Reviewer #2:**

*We thank the reviewer for their time reviewing our manuscript and we appreciate that this reviewer notes that the work merits publication in PLOS One. We have read the feedback carefully and have implemented their suggestions, which we believe address all the concerns raised.*

In the manuscript titled “An automated high-content screening and assay platform for the analysis of spheroids at subcellular resolution” the authors present the development of a technique to generate a large number of arrayed relatively uniform spheroids using micropatterned multi-well plates. The authors describe how these samples can be imaged using an automated high-resolution image cytometer and analyzed at the spheroid-, cell-, and subcellular-level using an algorithm developed within the Harmony software platform. Finally, they demonstrate how the assay can detect changes induced by inhibitors that affect the Golgi apparatus or microtubules. The strength of the manuscript is the presented techniques address an ongoing need in the research community to efficiently generate and analyze more representative 3D samples. The key weakness is the limited characterization of the quality of generated spheroid. Although the manuscript would be significantly strengthened if this weakness was addressed, the work presented in the manuscript is well written and scientifically sound and merits publication in PLOS One.

Strengths

1) The authors introduce a method to generate large numbers of relatively uniform spheroids. If proven broadly applicable, this would advance the ability to perform HTS using spheroid samples.

2) The authors demonstrate how tools within the Harmony software platform can be used to achieve sub-cellular analysis resolution.

Weakness

1) It is unclear if this technique is producing quality spheroids with cells tightly interacting with each other or simply cell aggregates centered on the micropatterned ‘well’. The manuscript would be strengthened significantly if efforts were made to assess the quality of the spheroids produced by the micropattern technique. Alternatively, authors could demonstrate how responses differed in the 3D spheroid vs the 2D culture of the cell lines used in the study, which would indicate biological differences associated with the 3D samples.

*The focus of this manuscript is technical in nature (rather than presenting biological results from a screen, or making a direct comparison between 2D- and 3D-grown cells), detailing an approach that we believe has the potential to facilitate high-resolution imaging from a small spheroid model in an automated screening format. Our initial optimisation experiments and associated calculations suggest that the spheroids all form from the initial cells that are deposited on the micropattern directly following cell seeding. Unfortunately, we are a little unsure what ‘quality’ measure this reviewer is requesting. We believe that this is the most detailed quantitative characterisation of spheroids presented to date, providing information on sphericity, volume, surface area, and number of nuclei. Interestingly, these values are comparable to those measured in small spheroids generated using more ‘conventional’ production methods, for example growing them in extracellular matrix (ECM) basement membrane material (Ref. no. 41). This suggests that their characteristics (quality) are similar to spheroids formed using established methods.*

2) The focus of the manuscript is somewhat unclear.

*We have made this manuscript more technical / methodological in focus, by providing more detail in the Materials and Methods section and Appendices (lines 140-157, 175-185, 195-206, 233-238). Importantly, we now provide the detailed image analysis pipelines used to generate the various quantitative results, enabling others to replicate our experiments.*

2a) It appears written primarily as a technique/assay development paper. As such, further detail on the methods/analysis algorithm used could be provided. The optimization work would be important to share in supplemental material. Importantly, it should be made clear if the Harmony algorithm will be made/are available to the research community. In addition, metrics describing assay quality (for example, Z’-prime values) should be calculated and provided.

*Please refer to the response above. We have made all the analysis pipelines available as tables in the Appendix. The Harmony software is proprietary software from Revvity and we are unable to distribute it. However, in other work we have previously shown how image analysis pipelines generated in Harmony can be replicated in the open-source software Cell Profiler (Garcia-Pardo et al., 2021; PMID:34496765). It is not possible to calculate Z’ values for the data shown in this work. Z’ values are used in screens to compare experimental values to positive and negative controls, and thereby determine the screen quality. However, in the work that we present, there are no positive controls as such; rather the approach details how strengths of phenotypes can be compared. It should also be noted that Z’ values can only be applied to data that show a normal distribution; this also does not apply here.*

2b) The authors devote significant space describing the importance of using 3D cultures/spheroids to generate more representative samples to study cell physiology compared to 2D cultures, however, no data was presented to suggest the current assay captures that difference. The advantage of 3D spheroids are intended to be the focus of the manuscript, comparative data with 2D cultures should be included.

*In the revised manuscript, we have added significantly more methodological information to make this a technical paper (lines 140-157, 175-185, 195-206, 233-238). Carrying out a comparative screen with monolayer cells would be a manuscript in its own right and is far beyond the scope of this work.*

Other Comments

1. Line 73 – Missing punctuation/incomplete sentence

*We apologise for this error, caused by a change in formatting just prior to submission. The missing punctuation has been added and the incomplete sentence finished (see line 77).*

2. Line 165 – Methods list several objectives used, but it is unclear in the manuscript where each (if all) were used.

*Each figure legend details the specific objective used to generate the corresponding image data. This information can be found in Figure legends 1-4 and S2-S4. The 40x water immersion objective was not used in any of the data presented and its mention has been removed.*

3. Line 166 – Typo “63x/1.1.15NA”.

*This typo was corrected (see line 171).*

4. Line 189 – More accurate to state that statistical analysis of single cell data is not presented due to the large number of samples generating significant differences with limited/unclear biological meaning.

*Statistical analysis has now been performed for the data in Figures 3-5 and S2-S5. The figures have been modified to reflect this and information on how the statistical analysis was performed has been added (lines 211-214) and is in the relevant figure legends. It is important to note, however, that when comparing features for thousands of individual organelles from hundreds of cells, even small differences between values result in extremely small p-values that may be misleading. We have discussed this with an expert statistician and taken advice on how to analyse very large data sets such as this. The point about an unclear biological meaning is a good one, and is now included in the Discussion section (lines 526-530).*

5. Line 206-207 – As an assay development paper, it might be useful to describe the optimization process in greater detail for others attempting similar assays using alternative cell types.

*We agree with the reviewer on this point. We have added further comments on the optimisation process in the revised manuscript (lines 233-238).*

6. Figure 1 Legend – Would be good to indicate if displayed image represents a single field or a stitched collection of fields.

*A sentence has now been added to the figure legend saying that the images are stitched together from single fields of view (lines 264-265).*

7. Line 344-345 – For readers not familiar with the Harmony software, it would be good to provide the type of texture being measured by the ‘plane bright’ and ‘saddle’ texture components.

*The texture PLS (Plane, Line, Saddle) features used in 3D analysis are equivalent to SER (Saddle, Edge, Ridge) features used in analysis of monolayer (single slice) images. This information has been added to the Materials and Methods section (lines 201-206).*

8. The authors should comment about any issues with photobleaching when collecting a large z-stack at 100% laser power and image exposure times up to 500 msec.

*We did not observe any issues with photobleaching while imaging the spheroids under the conditions used. We were even able to re-image plates up to 2 weeks post-fixation without any significant loss of signal intensity. We have added a sentence in the Materials and Methods section to confirm lack of photobleaching (lines 183-185).*

9. It would be informative if authors provided an average scanning time per plate/per well to collect the low and high resolution images.

*The pre-scan of a full plate (60 wells) with the 20x water immersion objective took between 45-60 minutes. The rescan took between 6-9 h depending on the number of wells and number of spheroids chosen for the rescan. This information has been added to the Materials and Methods section (lines 181-183).*

10. A key limitation of high NA objectives is a limited working depth. While this did not impact the current study due to relatively small spheroids generated using the microprinting technique, it would be good if the authors commented on how this might impact broader application of the methods to other types of

*As pointed out by the reviewer, we specifically chose to use smaller spheroids to avoid this issue. Larger spheroids (up to 150 µm in depth / height) can be imaged successfully using this approach, still generating information with subcellular resolution. This comment has been added to the Discussion section (lines 489-495).*

11. Scale Bar length vs spheroid shown in figures. Figure 1D indicates an average spheroid width ranging between ~80-90 um. However, at a scale bar length of 50 um, it appears many of the spheroids shown at higher resolution in figures are approaching ~150 um width, falling within in the far extreme range observed at 20X. Confirm that scale bar length is correct, or it would be good to state in the text/figure legend that especially large spheroids were chosen to allow easier visualization of the phenotype/masking.

*We thank the reviewer for spotting this. Indeed, the scale bar was incorrectly annotated on the figure. The scale bar has been corrected, and a new Figure 2 uploaded.*
