# Supplementary material for: An automated high-content screening and assay platform for the analysis of spheroids at subcellular resolution
Source: PLoS One. 2024 Nov 12;19(11):e0311963. doi: 10.1371/journal.pone.0311963 (PMC11556727; doi:10.1371/journal.pone.0311963)
Supplement: Attachment — Submitted filename: Response to Reviewers.docx [file pone.0311963.s014.docx]

**Reviewer #2:**

*We now address the additional queries mentioned by this reviewer, which we note several of which were not raised in their original assessment of the manuscript.*

[Line 189] The authors state spheroids were segmented using the A647 secondary antibody signal. For any significant screening project, the use of antibodies would significantly increase the cost per well. Therefore, it would be beneficial if the authors could comment if similar spheroid region segmentation is possible using either the DNA dye or GFP signal (when present).

*In lines 189-191, we already stated in the previous version of the manuscript that spheroid-level segmentation based on either a plasma membrane stain or the Hoechst 33342 staining is possible. We refer the reviewer to lines 189-191 both in this version and the previous manuscript version.*

[General comment] The authors describe the developed tools as useful for screening, however, more effort could be made to describe how much variation observed between replicate samples. For example, well-to-well variation in the average number of detected objects per cell or per spheroid.

*This is an additional query not raised in the original review of the manuscript. We have analysed the numbers of spheroids detected between replicate wells and determined the coefficient of variance to be in the order of 5% between replicate wells. A comment to this effect has now been added (see lines 258-260).*

[Line 282] The use of the phrase "a high degree of accuracy" suggests the use of a metric that quantifies segmentation quality compared to an annotated ground truth set of images (i.e. IoU, F1, etc analysis). If no such comparison was performed. it may be better to make a statement stating that the assessment of accuracy was qualitative and not quantitative.

*This is an additional query not raised in the original review of the manuscript. The phrase “a high degree of accuracy” has been removed (see lines 284-285).*

[General Comment] Charts appear low resolution in the PDF. This is not an issue with the supplemental figures.

*All of the graphical data, throughout the manuscript, have been prepared in an identical manner and following the journal guidelines (300 dpi). We suggest to the reviewer that the low resolution of the graphs in the pdf is a consequence of how the pdf builder on the journal’s website assembles the document in a compressed format for peer review, and so this would not be expected to be an issue in the final published version.*

[General Comment] Instances where the authors' description of the data does not match what the figure shows. For example, authors describe figure 5A data (line 367) as 'no detectable difference in the number of structures', yet, the figure indicates a significant difference. This is a carry-over of the revision now containing the statistical analysis of single-cell data, but the language used need to be corrected.

*The choice of language to describe these changes has now been modified (see lines 311, 339-340, 363, 369-370, 413, 418). We also again refer the reviewer to lines 529-533 of the manuscript where we discuss the use and limitations of statistical tools when analysing large numbers of individual object data in high-content screens.*

[Line 732] The figure legend does not indicate the meaning of the colors used. Are the individual objects pseudo-colored?

*This is an additional query not raised in the original review of the manuscript. A sentence clarifying this has been added to the figure legend S1 (see line 738).*
